# Designing nature networks for cities: combining multi-species modelling approaches

**DOI:** 10.1007/s10980-026-02315-0

**Published:** 2026-03-01

**Authors:** Anna M. Bracken, Luca Nelli, Luigi Cao Pinna, Alistair Corbett, Rory McLeod, Davide M. Dominoni, Dominic J. McCafferty

**Affiliations:** 1 School of Biodiversity, One Health and Veterinary Medicine, University of Glasgow, Graham Kerr Building, Glasgow, G12 8QQ UK; 2 School of Biological, Earth and Environmental Sciences, University College Cork, Cork, T23 N73K Ireland; 3 School of Mathematics and Statistics, The Mathematics and Statistics Building, University of Glasgow, Glasgow, UK; 4 Council for Agricultural Research and Economics, Research Centre for Agriculture and Environment (CREA-AA), Via Della Navicella, Rome, Italy; 5 Glasgow City Region Green Network, Floor 2, Room 29, 40 John Street, City Chambers East, Glasgow, UK; 6 Scottish Centre for Ecology and the Natural Environment, School of Biodiversity, One Health and Veterinary Medicine, University of Glasgow, Rowardennan, Glasgow, G63 0AW UK

**Keywords:** Ecological connectivity, Grasslands, Pollinators, Expert-knowledge, Habitat suitability

## Abstract

**Context:**

Urban wildlife habitats are often fragmented and of poor quality, yet cities hold potential to support biodiversity, particularly for small-bodied species like insect pollinators. Enhancing habitat connectivity is essential for improving biodiversity and increasingly prioritised in planning frameworks. Combining diverse approaches to assess habitat connectivity may yield the greatest overall success.

**Objectives:**

We compare two multi-species modelling approaches for assessing urban ecological corridors. The first species-specific approach uses combined habitat suitability maps of four insect pollinators and assesses connectivity using resistance modelling (circuit theory). The second landscape-level approach has been developed by urban environmental planners (“Green Network Development officers”) and identifies core areas as species-rich habitat patches using spatial data, species records (of plant and pollinator indicator species), and local expertise, then models connectivity between these using least-cost paths. By comparing these two approaches, we aim to identify gaps and priority areas for habitat creation or management.

**Methods:**

Using biological records, we mapped habitat suitability for pollinators and applied circuit theory to assess connectivity and identify “pinch points”—bottlenecks to movement that can be targeted for corridor enhancement.

**Results:**

While both approaches showed 39 km^2^ of overlap, 31 pinch points—often centred around core pollinator habitat—were outside the corridors identified by Green Network Development officers. These areas could be prioritised in future iterations of the ecological network.

**Conclusions:**

Our species-specific modelling approach identified 31 pinch points outside of planner-defined corridors, highlighting important areas of movement constraint not captured by the current planning framework. Incorporating species-specific modelling into urban planning also helps identify key habitat variables impeding movement, enhancing the biological understanding of the system. We recommend urban planners adopt multiple, complementary approaches for corridor delineation and collaborate with researchers, ecologists, and citizen scientists.

**Supplementary Information:**

The online version contains supplementary material available at 10.1007/s10980-026-02315-0.

## Introduction

Urbanisation is accelerating at an unprecedented rate, driving global biodiversity declines. To address this crisis, the Kunming-Montreal Global Biodiversity Framework, adopted at COP15, set ambitious global targets to achieve a vision of living in harmony with nature by 2050 (CBD 2022). Central to this is the “*30* × *30” target*, which commits more than 100 countries to protect 30% of land and sea by 2030 (WWF and IUCN WCPA 2023). Delivering on this target will vary between countries, as governments must translate global goals into actionable strategies, tailored to specific landscape contexts and associated challenges. A widely recognised strategy is the enhancement of ecological connectivity, which enables wildlife to move freely between habitats, thereby improving genetic diversity and ecosystem resilience (LaPoint et al. 2015). For example, in Scotland, the 30 × 30 target is to be delivered through the “Nature Networks Framework”, which aims to connect urban and rural landscapes via wildlife corridors and stepping-stones (NatureScot 2024). However, despite their potential, designing effective ecological networks is complex, as there is no universal method for assessing connectivity or creating corridors to accommodate multiple species and habitats (Wood et al. 2022). These challenges are even more pronounced in urban settings, where human activity and competing land demands tend to be greatest (LaPoint et al. 2015; Lookingbill et al. 2022). Combining approaches for multi-species ecological connectivity assessment may enhance the resilience and functionality of urban wildlife corridors (Zeller et al. 2012).

Wildlife corridor design will vary by each location, as well as by the methods employed. Broadly, multi-species connectivity assessments can take either an “upstream” or “downstream” approach (Wood et al. 2022). Upstream approaches prioritise environmental features or use “generic” species traits to infer landscape-level connectivity (Sanderson et al. 2002; Koen et al. 2014; Marrec et al. 2020), while downstream approaches focus on calculating species- or group-specific connectivity, combining resulting analyses later in the process. As a result, upstream methods typically provide a broader overview of landscape connectivity, whilst downstream approaches can be more realistic for certain species or groups; though downstream methods are typically more computationally intensive due to species-specific modelling (Wood et al. 2022). Combining results from different methods can offer a complementary overview of ecological connectivity within a region (Krosby et al. 2015). To parameterise connectivity models, regardless of the approach, both *nodes* (habitat patches between which connectivity is assessed) and *resistance layers* (a measure of ease of movement through the landscape) need to be assigned. This can be achieved through various techniques, such as assigning costs to habitat maps using expert knowledge (Urbina et al. 2024), or modelling habitat suitability for focal species by combining geolocation and environmental data and using this as a surface to compute ecological connectivity (Cianfrani et al. 2013; Mohammadi et al. 2022). The latter method can give finer information on species-specific habitat preferences and movement (Nelli et al. 2022). Many techniques for computing connectivity exist, but commonly used methods include least-cost modelling (Adriaensen et al. 2003) and circuit theory (McRae et al. 2008). The former determines a continuous path with the lowest ‘cost’ to an organism moving between habitat patches and assumes an animal has complete knowledge of the landscape (Etherington 2016). The latter models animal movement as a random walk across a two-dimensional landscape conductivity surface (McRae et al. 2008), and has been used to identify areas of high connectivity potential that may not be immediately apparent from least-cost paths alone. Advancements in these techniques have been supported by the growing availability of species location data through citizen science recording schemes, which represent an important data source, particularly in urban areas (Bradsworth et al. 2017; Nelli et al. 2022; Turner et al. 2022; Chen et al. 2024; Fung et al. 2024).

Pollinating insects (hereafter ‘pollinators’) are a key focus for meeting global biodiversity targets, as they provide essential ecosystem services, but are undergoing widespread declines (Nath et al. 2023). Urbanisation tends to have contrasting trait and scale-dependent effects on pollinators but typically causes declines in diversity where impermeable surfaces reach high levels (> 50%) (Wenzel et al. 2020). Recent studies emphasise the importance of plant species richness in mitigating negative urban effects, as pollinators respond to the availability of local flowering plants, often irrespective of the broader landscape (Liang et al. 2023). In this respect, urban settings have the potential to serve as important habitat for more generalist pollinator species (particularly bees: Theodorou et al. 2020), especially as surrounding agricultural areas are often associated with reduced pollinator diversity (Seibold et al. 2019). However, habitat fragmentation is a major driver of pollinator decline, making ecological connectivity a critical factor for species persistence in urban environments (Wagner et al. 2019; Zhang et al. 2022). Urban grasslands—a key habitat for pollinators (Dylewski et al. 2019; Buchholz et al. 2020)—are particularly at risk of intensive management, development and fragmentation (Klaus 2013; Ignatieva et al. 2020; Fekete et al. 2024). For pollinators, which rely on fine-scale connectivity between gardens, green spaces, and remnant natural habitats, maintaining functional habitat linkages is essential not only for their persistence but also for wider regeneration of local ecosystems. Despite this, ecological assessments for pollinators remain underrepresented in the literature, particularly in urban contexts (LaPoint et al. 2015; Lookingbill et al. 2022). Pollinator response to fragmentation differs from other taxa due to variation in life history traits, foraging ranges and movement capacity (Viana et al. 2012; Jauker et al. 2013), and therefore corridors designed for larger taxa may not be suitable. As this is a relatively new area, using different, but complementary techniques to assess multi-species connectivity for pollinators is likely to lead to improved conservation outcomes.

Here we compare two approaches for assessing multi-species habitat connectivity in a major city, that of Glasgow and the surrounding areas. The two approaches are based on differing premises—the first a “downstream” method focussing on species-specific connectivity, and the second an “upstream” method, focussing on habitat patch connectivity—so that comparison will help identify priority areas to target to enhance functional ecological connectivity. The first approach estimates species-specific connectivity using biological records to model the habitat suitability of four generalist pollinators: White and Buff-tailed bumblebees (*Bombus* species, grouped due to ambiguity in species identification and overlap in habitat preferences), Meadow brown butterfly (*Maniola jurtina*), Ringlet butterfly (*Aphantopus hyperantus*) and Small heath butterfly (*Coenonympha pamphilus*). These species were chosen due to their generalist life histories and broad habitat use (e.g., grasslands, meadows and woodland), ensuring that resulting corridor networks are widely applicable. Habitat suitability maps are combined, and overall collective connectivity is assessed using circuit theory. The second approach has been developed by urban environmental planners (Green Network Development Officers; Glasgow City Region Green Network, GCRGN: GCR Green Network 2025) in partnership with Glasgow City Council and seven other local authorities that make up Glasgow City Region. The aim of GCRGN is to prioritise targeted habitat deliverables in Glasgow, focussing on urban grasslands (and other opportunity areas) to increase connectivity for pollinators and other wildlife as part of a Grassland Nature Network. In brief, the GCRGN approach involved four main steps: (1) identification of core areas as species-rich grassland patches using expert information, biological records of indicator plant and pollinator species, and existing spatial datasets; (2) modelling habitat networks between core areas using least-cost techniques and generic pollinator dispersal distances; (3) opportunity mapping for targeted habitat creation; and (4) development of draft grassland nature networks through expert interpretation of model outputs and correlation with additional spatial data (on green space, open space audits, golf courses, protected sites, the National Health Service (NHS) estate and linear infrastructure) to identify potential habitat delivery opportunities.

Combining connectivity approaches has been shown to effectively identify critical corridors and connectivity pinch points—key areas essential for reducing pollinator population isolation—between the most highly suitable habitats (Kwon et al. 2021). GCRGN draft Nature Networks have been defined after rigorous input from stakeholders and experts with local knowledge of sites. Our objective therefore was to use a different species-specific approach to compare with selected corridors. We assess corridor overlap, identifying areas of agreement (where the creation of a corridor is likely to improve functional connectivity) and areas of discrepancy (where there may be potential for further improvement of connectivity). Finally, we identify connectivity pinch points and compare these to GCRGN-defined corridors to prioritize critical areas for conservation efforts. We expect that our species-specific approach will be more fine-tuned to local environment and therefore highlight areas of critical importance to the pollinator network that may or may not be picked up by the GCRGN corridors. By comparing diverse assessments of wildlife corridors, we aim to evaluate their robustness and identify priority areas for enhancing functional connectivity.

## Methods

### Study area

We conducted our study across the City of Glasgow plus a 10km buffer, covering council areas of Renfrewshire, East Renfrewshire, West Dunbartonshire, East Dunbartonshire, Stirling, North Lanarkshire, South Lanarkshire, and East Ayrshire. We chose a 10km buffer to minimise edge effects in connectivity analyses (Koen et al. 2010) rather than an area reflective of species mobility, since dispersal distances for our study species are relatively low (Table S1). Note that the GCRGN Clyde Grasslands connectivity map was originally generated for a larger area (see: GCR Green Network 2025) but was restricted in these analyses for comparison with the species-based connectivity approach.

The study region is home to over one million people, 42% of whom live within the Glasgow city council boundary. However, Glasgow city also encompasses much greenspace, as over a fifth of the city’s total area is designated greenspace (Glasgow City Council 2024a).

### Modelling approach 1: species-based connectivity modelling

#### Occurrence data

**Fig. 1:**
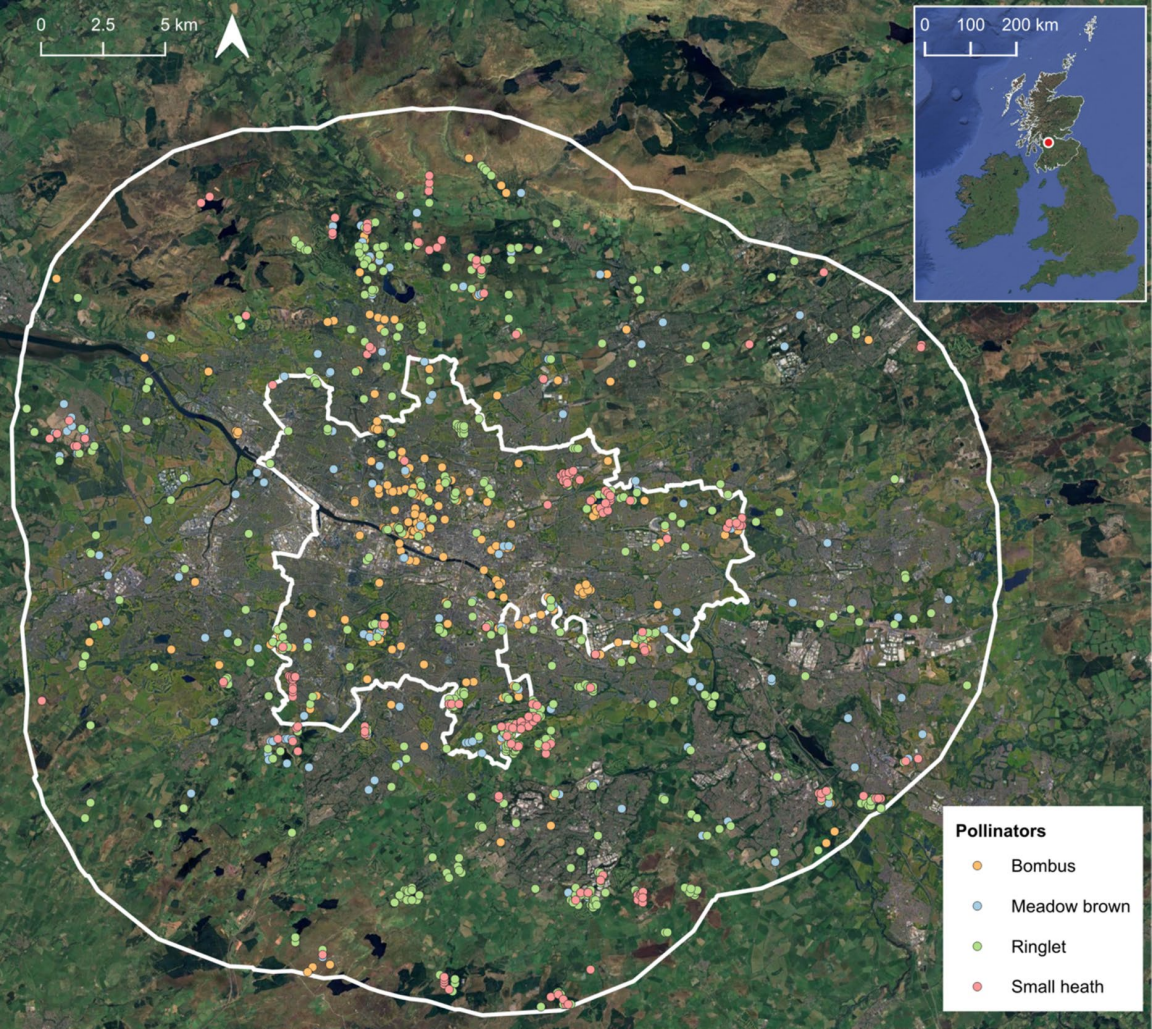
Distribution of pollinator presence records in Glasgow city and surrounding areas. Inner white polygon is Glasgow City Council boundary and outer white boundary the 10km buffer. Inset: position of study site (red point) in Scotland, UK. Records were subset for between 2015 and 2022. Duplicates and records with > 100m uncertainty in spatial position were discarded

We use wild pollinator species occurrence data collated and verified by Glasgow Museums Biological Records Centre (GlasgowLife 2024), which was provided on 15/02/2023. Occurrence data consisted of biological records, most of which arose from citizen science, both opportunistic (from individual submissions to online recording schemes like iRecord and iNaturalist) and more standardised surveys (such as national biodiversity initiatives like the UK Butterfly Monitoring Scheme and the Big Butterfly Count) (Weddle and Haggard 2024). We selected four candidate species with sufficient data from the two main wild pollinator groups in the UK: bumblebees (White and Buff-tailed *Bombus spp.*) and butterflies (Meadow brown: *Maniola jurtina*, Ringlet: *Aphantopus hyperantus*, Small heath: *Coenonympha pamphilus*) (Fig. 1). These species were chosen due to their generalist life histories and use of a variety of habitats. However, we modelled their habitat suitability separately, as each species has slightly different requirements. White and Buff-tailed bumblebees were grouped due to species ID ambiguity in biological records, but also because both species are synanthropic; sharing similar habitat and foraging preferences in urban landscapes (Lubiarz and Trzaskowska 2013), using flowering plants in residential gardens or parks, as well as scrubland and woodland clearings (Foster et al. 2017). Meadow brown butterflies thrive in a range of habitats but typically select for long grass (Pywell et al. 2004), Ringlet butterflies are more commonly found in damp, shaded woodland (Warren 1985), while Small heath favour dry areas with fine grasses (WallisDeVries and Ens 2010). Assessing habitat suitability separately allows us to model and retain species-specific habitat preferences and then combine to use as a basis to compute overall connectivity.

We subset data to include records from 2015 to 2022, aligning with the years for which environmental variables were collected and when the majority of records were submitted (see Fig S1), providing seven years of occurrence data. We discarded records with a coordinate uncertainty greater than 100m (the coarsest resolution of our environmental variables; removing 183 records or 5% of the total dataset), excluded duplicate entries and records with unknown dates, resulting in a final dataset of 1,591 observations (*Bombus*: n = 390; Meadow brown: n = 392, Ringlet: n = 633, Small heath: n = 176; Table S1).

To mitigate against observation bias—where records are more common in accessible or populated areas (Phillips et al. 2009)—we selected background records using kernel density estimation, which weights background points towards areas with a high density of presence records. This method has been successfully applied to citizen science data in previous studies (Deshwal et al. 2021; Nelli et al. 2022; Cao Pinna et al. 2024). We selected background points at ten times the number of presence records.

#### Environmental predictors

To examine the influence of habitat features on the probability of insect presence, we selected candidate environmental predictors relating to land use, climate and terrain, and human pressure. We obtained land use variables from UK Centre for Ecology & Hydrology 10m resolution maps (Marston et al. 2022) and Ordnance Survey data (Ordnance Survey 2024). We extracted the proportions of key land cover types within a 100m radius: woodland (broadleaf and conifer), wetland (fen, marsh, saltmarsh, swamp, bog), arable, and grassland (neutral, calcareous, acid, improved, heather and shrub). We also extracted the proportion of urban greenspace types considered important for pollinators—private gardens and allotments/community gardens—within the same 100m radius (Baldock et al. 2019; Baldock 2020). Private gardens may provide improved habitat in urban landscapes (Sperling and Lortie 2010), while allotments and community gardens may offer additional resources, as they often host pollen-rich weed species and flowering fruits and vegetables (Baldock et al. 2019; Daniels et al. 2020).

We further calculated the proportion of impervious cover (a combination of buildings and roads with a 5m buffer) within a 100m radius, as this has been shown to reduce pollinator abundance (Geslin et al. 2016). Additionally, we assessed human pressure using human population densities at the output area level (the smallest geographical unit for census data) from National Records of Scotland 2022 population maps (National Records of Scotland 2024), as higher human densities have been associated with lower pollinator abundance (Persson et al. 2020).

We converted greenspace, impervious cover, and human population vector files to raster format with a 5m resolution to retain fine-scale spatial information. Finally, we used a digital terrain model at 50m resolution to calculate altitude. Human population density and altitude were mean averaged for 100m radius around points. Therefore, the resulting model was at a resolution of 100m. We standardised all variables by subtracting the mean and dividing by the standard deviation for effect size comparison.

#### Habitat suitability and connectivity modelling

We used binomial (logit link) generalised additive models (GAMs) to estimate habitat suitability for each of four pollinator species. We used species presence (1) and absence (0) as the response variable, and environmental covariates (see Table 1) as predictors, which were included as smoothed terms with up to four degrees of freedom in the model (Randin et al. 2006; Meynard and Quinn 2007). We square root transformed mean altitude within 100m to approximate normality. We fitted models using the ‘mgcv’ package in R (Wood 2011) with restricted maximum likelihood, and corrected for the presence/absence imbalance (where absence data was 10 × more frequent than presence) by equally weighting presence and pseudoabsence data, as recommended by Barbet‐Massin et al. (2012). We included a smoothed spatial term for x and y coordinates to account for spatial autocorrelation in the data. Human population density, proportion of impervious surfaces and proportion of gardens were highly correlated (Spearman’s rank correlation > 0.7, Table S2). Comparing each predictor in separate models produced similar fits (ΔAIC < 5, Table S3). We therefore chose to retain only proportion of gardens since it is a direct measure of green habitat providing foraging and nesting resources for pollinators, it captures heterogeneity in habitat quality not distinguished by impervious surfaces or population density, and is an actionable metric for urban conservation and management. We started with a full model considering all variables (Table 1) and selected predictors by “shrinkage” of all smoothed terms, using the “double penalty shrinkage approach” (Marra and Wood 2011).

**Table 1:** List of candidate environmental predictors used for modelling the habitat suitability of pollinators across Glasgow city and surrounding area

Source	Variable	Description	Values
UKCEH & Ordnance Survey	wood wet arable grass garden allot imperv	Proportion of woodland within 100m Proportion of wetland within 100m Proportion of arable land within 100m Proportion of grassland within 100m Proportion of private gardens within 100m Proportion of allotments within 100m Proportion of impervious surfaces within 100m	0–1 0–1 0–1 0–1 0–1 0–1 0–1
50m DEM	alt	Mean average altitude within 100m	0–520.90
Office of National Statistics	popdensity	Mean average human population density (people per ha) within 100m	0–482.60

To validate the selected model for each species, we performed both random and spatially blocked 10-fold cross-validation, allocating 75% and 25% of the original data for training and testing, respectively. Random cross-validation was used to assess model discrimination under our interpolation objective, as prediction areas were within the same geographical space in which our data were collected, and training and prediction areas were therefore not independent (Milà et al. 2022). To account for potential spatial dependence in the data, which can cause random cross-validation to overestimate predictive performance, we additionally implemented spatially blocked cross-validation using the “blockCV” package in R (Valavi et al. 2018). Spatial blocks of 5km were used, corresponding to the average range of autocorrelation in the predictor variables. This approach enforces geographic separation between training and test data and provides a more conservative assessment of model performance. As expected, spatial blocking also resulted in less balanced folds, particularly given the strong class imbalance in the data (where pseudoabsences were 10 × the number of presences). Consistency in model performance across both validation approaches supports the robustness of the predicted suitability surfaces used in subsequent connectivity analyses.

We evaluated model performance using the test subset by calculating the area under the curve (AUC) receiver operating characteristics (ROC), the Boyce Index, and the true skill statistic (TSS), which all provide complementary information on predictive accuracy. AUC provides an overall measure of model discrimination, where values of 0.5 indicate model discrimination is no better than random, and values of 1 indicate perfect discrimination of validation data (Anderson et al. 2016). The Boyce Index assesses the frequency with which presences are classified as true and is widely used in cases where a model is built without real absences (Hirzel et al. 2006). It ranges between -1 and 1, where values around zero mean the model is no different than random, while values close to 1 indicate perfect discrimination (Hirzel et al. 2006; Jiménez and Soberón, 2020). TSS measures the difference between the true positive and false positive rate (sensitivity + specificity—1), based on a binary classification of model predictions; here we used the threshold that maximises the model TSS. TSS values range from −1 to 1, where 0 is discriminating at random, while values higher than 0.4 indicate a good model fit (Guisan et al. 2017). We averaged performance measures across all folds and derived model estimates from the initial model. Importantly, AUC and the Boyce Index are threshold-independent metrics that primarily assess how well models rank locations along a suitability gradient (i.e., whether presences tend to receive higher predicted values than background points), whereas TSS is threshold-dependent and evaluates model performance at a single cut-off.

We predicted model estimates for each pollinator onto the entire study area to create individual habitat suitability maps. We summed these into a combined pollinator habitat suitability map and normalised to a scale between 0 and 1. The final combined map was highly correlated with individual maps (Pearson’s correlation mean ± SD: 0.89 ± 0.04) and therefore retained species-specific habitat suitability information. We used this grouped approach rather than computing connectivity separately, as grouping ecologically similar species into functional groups has been shown as effective in multispecies connectivity assessments (Brodie et al. 2015), and also greatly reduces computational demand. Given all four pollinator species were generalists with similar habitat requirements, the combined approach provides a generalised estimate of pollinator connectivity while retaining some species-specific information.

We used Circuitscape (McRae et al. 2008) to model connectivity. We used the inverse of the combined habitat suitability map as a measure of movement cost, and defined nodes as “pollinator core habitat”: groups of two or more adjacent cells with a combined and normalised habitat suitability ≥ 0.5 (Osborne and Suárez-Seoane 2007; Nelli et al. 2022) being within 300m of other grouped cells ≥ 0.5. A 300m cut-off was chosen as the smallest maximum dispersal distance among our focal species (Table S1). Habitat suitability thresholds were selected following a sensitivity analysis (Table S4; Fig. S2), which showed that on average a threshold of 0.5 maximised the balance between model sensitivity and specificity (maximising the sum of sensitivity and specificity, *maxSSS*, and minimising the sensitivity–specificity difference, *MDT*: Jiménez-Valverde and Lobo 2007; Liu et al. 2013).

To further examine the important areas which could constrain pollinator movement, we identified potential “pinch points”— areas where corridors narrow, creating natural bottlenecks or movement congestion (i.e., areas of suitable habitat which are relatively isolated: Sütünç, 2021). For this analysis, we used the PinchPoint mapper tool in ArcGIS to identify the narrowest sections within least-cost corridors between critical core areas (habitat suitability > 0.9). We selected this higher threshold to focus on the most confident areas of pollinator habitat to complement the main connectivity analysis (above), highlighting areas where connectivity is most vulnerable to disruption. This method firstly creates least-cost corridors using Linkage Mapper, then identifies pinch points within them using circuit theory (Circuitscape;McRae and Kavanagh 2011). We defined pinch points as a group of two or more neighbouring cells that were in the top 1% of pinch point values (using function “patches” in the “terra” package, R: Hijmans et al. 2023).

### Modelling approach 2: GCRGN clyde grasslands network

The full methodology used to model draft Glasgow City Region Green Network (GCRGN) corridors is available at https://www.gcrgreennetwork.co.uk/publications/clyde-grasslands-brochure. The starting point for this assessment was the GCRGN Clyde Grasslands study, which involved the following steps: (1) identification of “core habitat”: potential species rich grassland (SRG) sites in and around Glasgow, (2) least-cost habitat network modelling between SRGs, and (3) opportunity mapping for targeted habitat creation. Here, we briefly outline steps 1 and 2 below which generated least-cost corridors that we compare with our species-based connectivity approach. A fourth step used the Clyde Grasslands study outputs to support the identification of draft Nature Networks.

#### (1) Identification of core habitat—species rich grasslands

Species rich grasslands (SRGs) were identified using a combination of local knowledge (liaising with local authorities and biological recorders), biological records as proxy indicators for habitat, and existing spatial datasets, from NatureScot (NatureScot 2025), UKCEH (Marston et al. 2022), Ordnance Survey (Ordnance Survey 2025) and directly from GCRGN. Records of indicator plant species from 2000 onwards (n = 50,121; 350 species) were obtained from the Botanical Society of Britain & Ireland (BSBI), and plant species richness was calculated for each 1km grid square (to assign ‘plant hotspots’). An ecologist cross-referenced these species-rich cells with aerial imagery and BSBI plant records to determine habitat parcels likely to contain an SRG (assigned as acid, neutral, calcareous or wet grassland). Clusters of indicator butterfly (Common blue *Polyommatus icarus*, Northern brown argus *Aricia Artaxerxes*, Small pearl-bordered fritillary *Boloria selene*) and moth (Grass rivulet *Perizoma albulata*) records (from year 2000 onwards, n = 1288) were obtained from Butterfly Conservation Scotland, to support habitat patch identification. Selected species are associated with species rich grasslands (with caterpillars feeding on indicator plants) and have relatively constrained dispersal. Existing spatial datasets were quality-checked (cross referenced with plant hotspots) and collated with other datasets to define SRG habitats. Site validation was carried out through consultation with a local expert (ecologist Dr Scott Shanks), along with cross-referencing a restricted list of indicator plant species. Finally, eight workshops with local authorities resulted in 146 further SRG areas being identified.

#### (2) Least-cost habitat network modelling

Least-cost modelling was undertaken in ArcGIS using SRUC’s Habitat Networking Tool, using a focal species approach. Source SRGs (core habitat between which connectivity was modelled) were defined as habitat patches equal to or above 0.5 ha, at least 22m apart, and not separated by a road. This resulted in 1,046 Source SRGs (≥ 0.5 ha) and 780 non-source SRG’s (< 0.5 ha). Local authorities also provided a “newly created meadows” layer. All layers were merged with a base habitat map (10m resolution), provided by NatureScot, which included land cover data, rivers, roads and railways. Each habitat type was assigned a cost representing its resistance to focal species movement (e.g., source habitats: cost 0, non-source SRGs and meadows: cost 1—which allows species to move freely). A cost of 2 indicates the focal species can only travel half of its dispersal distance. Network models were run for two dispersal distances: 800m (representing mobile species such as bumblebees) and 300m (representing less mobile species such as solitary bees). Final least-cost corridors were buffered on either side by the species’ dispersal distance, so the 300m network produces corridors 600m wide. Here we use this 600m Grassland Nature Network. Note this network was a draft at the time of this study.

### Corridor comparison

We performed a gap analysis (Rodrigues et al. 2004) to compare predicted habitat corridors from both approaches. Specifically, we compared areas of the highest predicted connectivity (the top 10% of all values) from the species-based connectivity approach to the GCRGN species-rich grassland corridors. We used additional pollinator records (the same four species) from the Global Biodiversity Information Facility (GBIF) collected after the original study period (2023–2025; GBIF.org 2025) to compare how well each corridor method predicted independent pollinator occurrence data. To do this, we calculated the proportion of independent GBIF presence data (n_presences = 430, mean ± SD across species: 108 ± 87) falling within four categories: (i) areas identified by both corridor types, (ii) species-based connectivity corridors, (iii) GCRGN corridors, and (iv) areas outside both corridor types. We then randomly generated 20,000 background points and calculated the proportion of absences falling in each of the four categories. We divided the proportion of presences by the proportion of absences to obtain the relative probability of pollinator presence per category.

We calculated descriptive statistics to understand *where* either corridor was predicted to occur: for non-overlapping areas for each corridor type (species-based corridors and GCRGN corridors) we extracted distance to the city centre (distance of grid cells containing a corridor to Glasgow city centre, extracted from “Glasgow_towncentres” shapefile: Glasgow City Council 2016) and proportion of dominant landcover classes in either corridor type. Finally, we compared the results of the species-based connectivity pinch point analysis with the GCRGN Clyde grassland corridors, to identify additional overlaps and gaps where connectivity may be at risk of disruption.

## Results

### Species-based connectivity modelling approach

Habitat suitability models showed good predictive performance under AUC (mean ± SD Random CV: 0.79 ± 0.04; spatial CV: 0.71 ± 0.01; Table S5), and Boyce Index (Random CV: 0.92 ± 0.04; spatial CV: 0.64 ± 0.11). TSS scores indicated marginal discrimination (Random CV: 0.45 ± 0.07; spatial CV: 0.32 ± 0.04). Important variables across all species included proportion of woodland and wetland. Proportion of gardens was predictive of pollinator presence for *Bombus*, Ringlet and Small heath species. Proportion of grassland predicted presence for *Bombus*, Meadow brown and Ringlet species. Altitude was a significant predictor for Ringlet presence, while the proportion of arable land was a significant predictor for Meadow brown presence. Proportion of allotments was not a significant predictor for any species. Full model outputs are provided in Table S6.

**Fig. 2:**
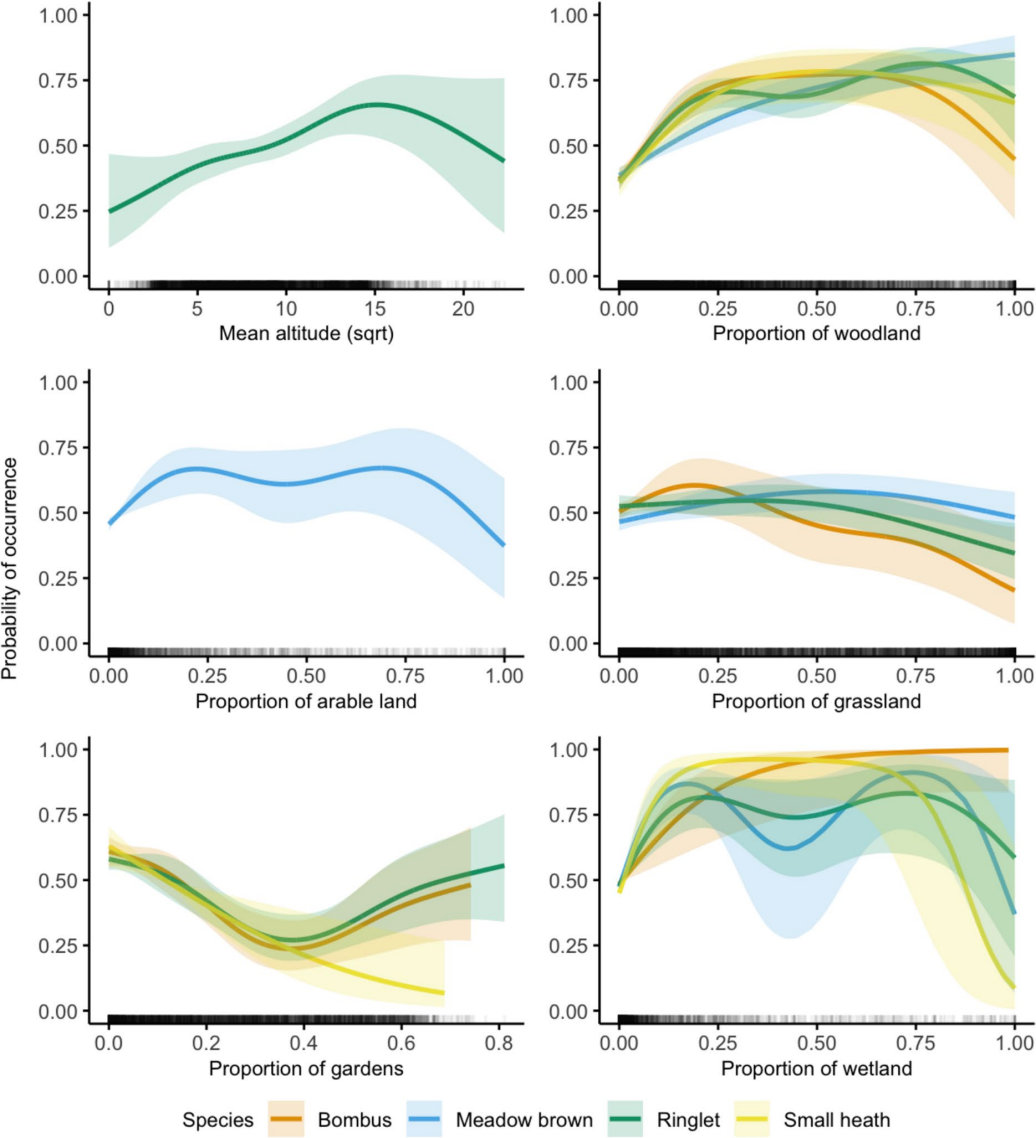
Significant environmental variables predicting the probability of pollinator presence (*Bombus*, Meadow brown, Ringlet and Small heath), from generalised additive models. Solid lines represent predicted values and shaded areas the confidence intervals. Where a species is not shown, the predictor was non-significant

Predicted pollinator presence showed a nonlinear relationship with all variables (Fig. 2). All species were predicted to occur in wetland habitats, though their responses varied: *Bombus* species presence was consistent across all proportions of wetland habitat, whilst other species showed declines when wetlands exceeded 75% of the area. Pollinator presence generally increased with the proportion of woodland, even in less densely wooded areas. However, *Bombus* species presence decreased in very densely wooded areas (woodland cover > 75%). Meadow brown, Ringlet and *Bombus* species occurred across all levels of grassland cover, though *Bombus* and Ringlet showed a steeper decrease as proportion of grassland increased.

Proportion of gardens had varying effects: *Bombus* and Ringlet species were predicted to be present in areas with either very low or very high proportions of gardens, whereas Small heath butterflies declined in areas with greater garden cover. Ringlet butterflies were more likely to occur at higher altitudes, while Meadow brown butterflies were associated with moderate proportions of arable habitats, but their presence dropped as these increased to > 75%.

#### Habitat suitability maps

**Fig. 3:**
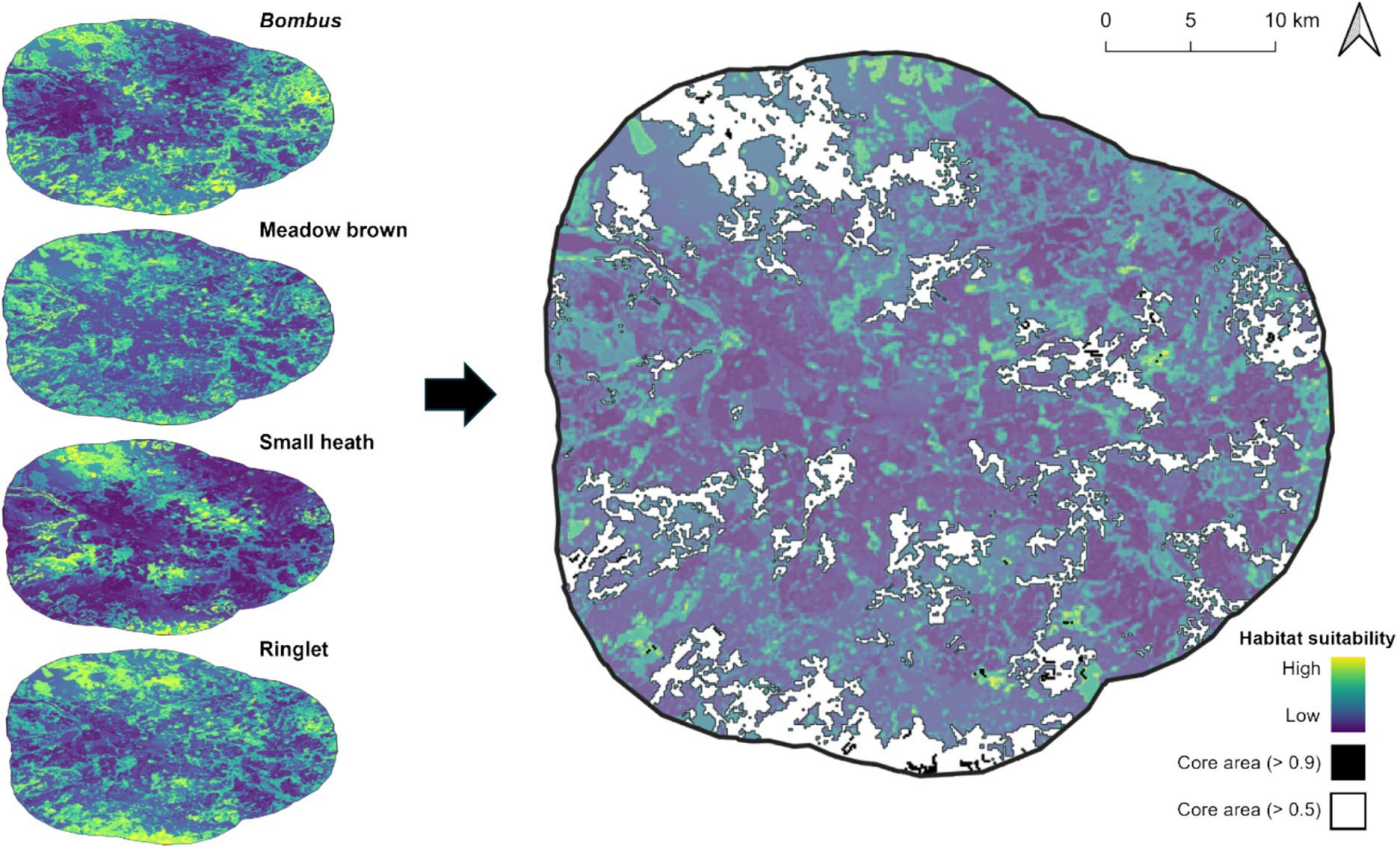
Individual (left) and combined (right) maps of pollinator habitat suitability. Individual maps were summed and normalised for the combined map. Low–high values of habitat suitability are indicated from blue–yellow, core areas (> 0.5) are shown in white with a black outline and critical core areas (> 0.9) are shown in solid black

We generated species-specific habitat suitability maps by applying the models to a 100m grid covering the study area (Fig. 3). These maps were then summed for each species and normalised to produce an overall habitat suitability score. Core areas, defined as having a normalised habitat suitability > 0.5, covered 214 km^2^, or 15% of the total study area. Raising the core area threshold to 0.9 reduced the core area to 2.5 km^2^ (0.17%), while largely retaining the overall habitat distribution.

### Corridor comparison

**Fig. 4:**
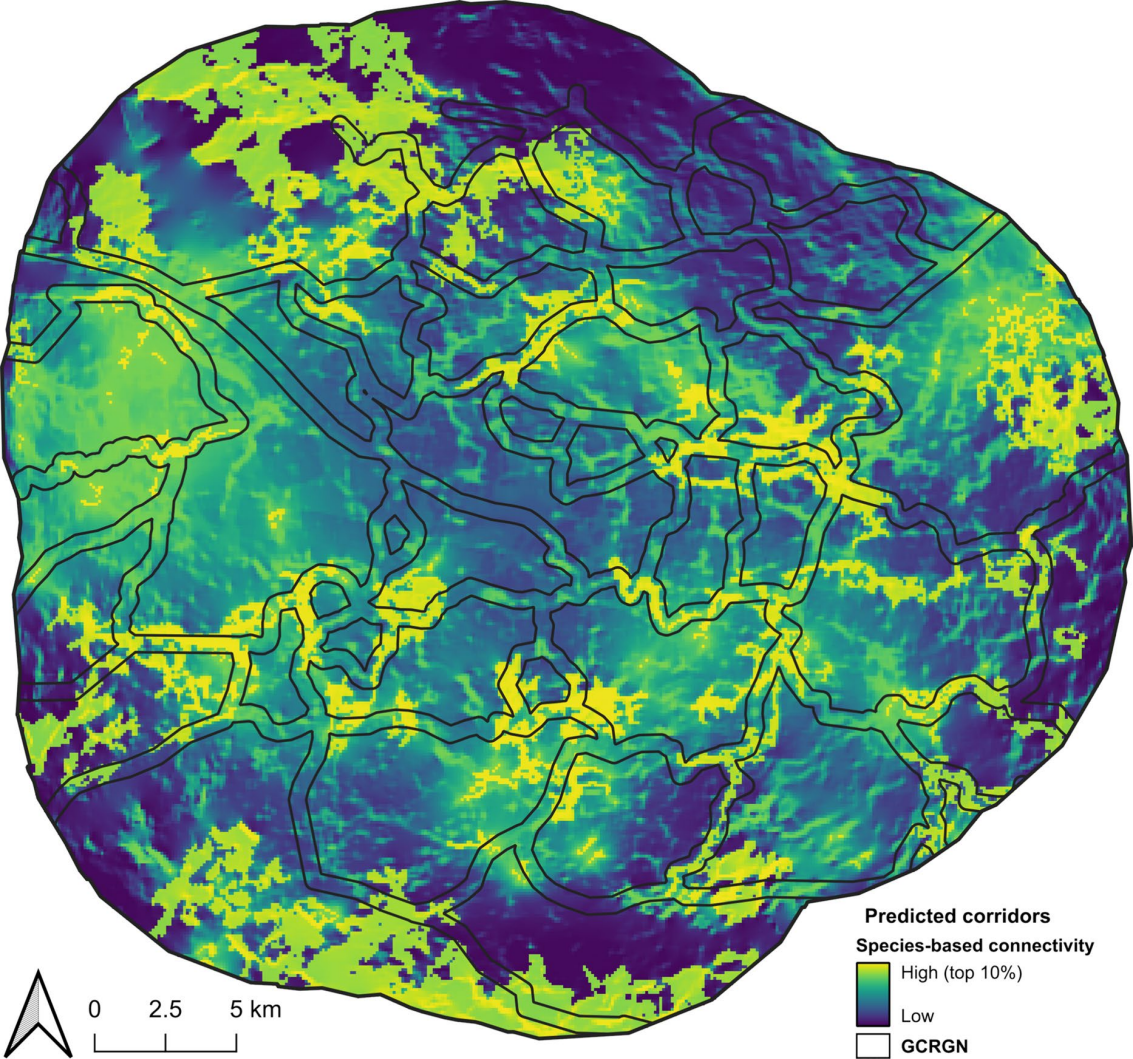
Predicted wildlife corridors from two multi-species modelling approaches in Glasgow, UK. Species-based modelling approach derived from habitat suitability and circuit theory is indicated from blue–yellow (low–high connectivity; top 10% of values are indicated in yellow). GCRGN corridors determined from least-cost paths between species-rich grassland patches are depicted with a black outline

Corridors showed an overall overlap of 39 km^2^ (Fig. 4). Corridors estimated from species-based connectivity approach (top 10% of values) covered 115 km^2^ (8% of the study area), whilst those from the GCRGN approach covered 247 km^2^ (17% of the study area). The species-based connectivity approach identified 77 km^2^ of predicted pollinator connectivity outside the corridors mapped by GCRGN, primarily in the north-west of the study area. Conversely, 208 km^2^ of corridors identified from GCRGN approach were not captured by the species-based connectivity model. Comparison of corridors using independent GBIF data indicated that records were more likely than random to fall in both corridor types (Fig. S3). Records were similarly likely to fall in either corridor type, with a slightly higher relative probability for GCRGN corridors (relative probability: both: 2.168; species-based corridors: 1.688; GCRGN corridors: 1.813). Presence records falling outside either corridor type were unlikely to occur (neither: 0.667). Species-based corridors typically contained greater amounts of woodland and wetland, whereas GCRGN corridors contained higher proportions of grassland, gardens, impervious surfaces and arable land (Fig. S4). GCRGN corridors were marginally closer to the city centre.

**Fig. 5:**
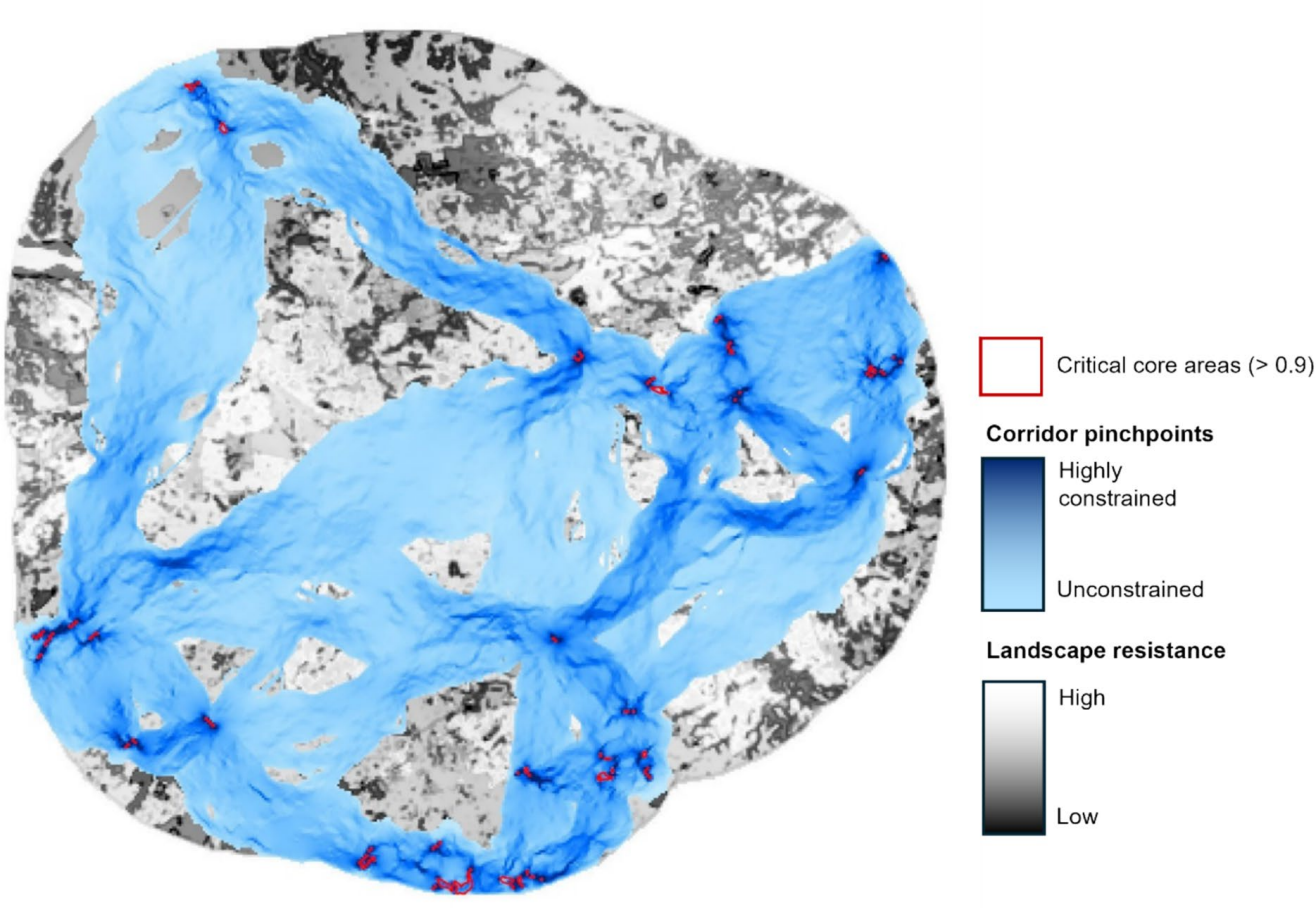
Pinch point analysis along least-cost paths derived from an inverted habitat suitability resistance layer of four pollinator species in Glasgow, UK. Constrained areas (pinch points) for pollinator movement are indicated from light-dark  blue (less to highly constrained). Landscape resistance is indicated from black-white (low-high resistance). Critical ‘core areas’ (> 0.9 habitat suitability) are indicated in red polygons

Pinch point analysis between critical core areas revealed 48 clusters (10.3 km^2^) likely to constrain movement, most of which were located near to core areas (Fig. 5).

Of the 48 identified pinch point clusters, 17 (2.4 km^2^) overlapped with GCRGN corridors, indicating the GCRGN network captures several areas of movement constraint. The remaining 31 clusters (7.9km^2^) fell outside GCRGN corridors, highlighting potential gaps where functionally important pinch points are not currently addressed (Fig. S5).

## Discussion

Ecological corridors identified through various methods are likely to be most effective in understanding species movement across urban landscapes (Peng et al. 2017). In this study, we highlighted overlaps and gaps between corridors identified from species-specific habitat suitability modelling and circuit theory, and those delineated by urban environmental planners (Green Network Development officers) using expert knowledge combined with biological records and least-cost models. Integrating the former, a conservative downstream approach based on species-specific habitat preferences, with the latter, a broad-scale upstream approach assessing landscape-level connectivity between species-rich grasslands, provides management bodies with priority areas for improving overall connectivity, ensuring conservation efforts are focussed and effective.

Both modelling approaches were derived from land-use variables, and therefore discrepancies arise from differences in how resistance layers and “nodes” (core areas of species habitat) were defined. While the Glasgow City Region Green Network (GCRGN) approach estimates connectivity between species-rich grassland patches verified by expert knowledge, the species-based connectivity approach models corridors from data on species-specific habitat preferences, that are not limited to grasslands. As a result, opportunity areas derived from GCRGN corridors may represent logistically feasible locations for habitat creation and enhancement (GCR Green Network 2025), whereas the species-specific approach incorporates a broader range of potential habitats, identifying key variables likely to impede species movement, thus providing a more comprehensive biological understanding of the system. Indeed, although our comparison uses the draft 600 m GCRGN nature networks, local authorities have since rationalised corridor widths to 500 m to align with other regional habitat corridors (woodland and wetland) rather than reflect species dispersal distances, highlighting their pragmatic approach to flagging habitat creation opportunities. Additionally, areas detected as “pinch points” derived from the species-based connectivity approach tend to represent smaller but potentially important habitat to target for improvement, which should be further validated with field data. Because ‘resistance’ and ‘node’ definitions differ, direct comparisons of model performance are not possible. However, both corridor types predicted independent data better than random, and therefore differences between models provide a more complete picture of landscape connectivity. An intuitive next step could be to combine methods—for example, by applying circuit-theory analysis to identify pinch points within GCRGN least-cost corridors. Moreover, combining techniques commonly used by managing authorities (expert-knowledge and least-cost modelling) with those often applied in academic research (habitat suitability connectivity modelling) fosters stronger collaboration, enhances understanding of key metrics, and ensures that the latest ecological insights are effectively incorporated into conservation planning (Karlsson and Bodin 2022).

Predictive performance of habitat suitability models varied with metric. AUC values indicate good discrimination across both validation methods, while Boyce Index values were lower for spatial cross-validation than random cross-validation. The very high Boyce values under random cross-validation may indicate overfitting and can be inflated by spatial clustering; however, the spatially blocked validation alleviates this concern. When training and test data were separated in space, Boyce values decreased but still indicated a non-random concentration of presences in higher suitability classes. TSS values were relatively low, potentially signalling only marginal discrimination; however, these values likely reflect the difficulty of defining a single threshold that simultaneously achieves high sensitivity and specificity under presence-pseudoabsence data with strong class imbalance, rather than an absence of useful discrimination along the suitability gradient. Given that our primary aim was to derive a continuous suitability surface to transform into a resistance layer for connectivity analysis, AUC and Boyce are more directly relevant to our use case than metrics based on a sharp binary “suitable/unsuitable” classification. We therefore interpret all three metrics jointly: AUC and Boyce support the use of the continuous suitability surfaces, while TSS is treated more cautiously as a diagnostic measure to contextualise threshold-based decisions for defining core habitat. Models revealed differences in species’ responses to environmental variables. Key habitats in urban settings—woodlands, wetlands and grasslands (Angold et al. 2006)—were important for generalist pollinator species. Indeed, both woodland and wetland predicted presence for all species. Woodlands and woodland rides provide essential habitats for butterfly pollinators, offering shaded areas and a grassy underlayer for caterpillar development (Alison et al. 2022). However, *Bombus* presence declined at over 50% woodland cover. While White- and Buff-tailed bumblebees use woodland glades, they prefer open grasslands with an abundance of wildflowers (Sikora et al. 2020) within range of their underground nests, reflecting their behaviour as central place foragers (Goulson and Osborne 2009). Instead, bumblebees had a strong positive response to wetland habitats, whilst other pollinator species showed more variable responses. Urban wetlands (e.g. fens, marshes, swaps and bogs) are important for bees, as they are often less developed in lowland areas, resulting in less intensive management (lower pesticide use) and more floral resources (Whitehorn et al. 2022).

Grassland cover predicted presence for all species except the Small heath, though a gradual decline in presence was observed at higher levels of grassland cover. Bee richness in urban grasslands is positively associated with ground-nesting availability and floral richness, while vegetation structure is important for butterfly species (Herrmann et al. 2023; Gordon and Kerr 2025). This result was unexpected; however, given the variability in urban grassland management, generalist pollinator species may shift to alternative habitats to access resources (Nielsen et al. 2014). Although human population density was excluded from the final models due to its high correlation with garden cover, it has been shown to reduce butterfly presence in urban habitats (Kuussaari et al. 2021) and future research could explore how areas of high human activity interact with grasslands to influence pollinator presence. Private gardens were significant predictors for all species except the Meadow brown. However, Small heath presence declined at high levels of garden cover, and both *Bombus* and Ringlet species indicated U-shaped relationships, where they were present at low (0%) and high (75%) proportions of gardens, but not in between. We do not have information on garden management, impervious cover at fine spatial scales or pollinator resource availability and therefore cannot determine the mechanisms underlying these patterns. Previous studies show that pollinator abundance and richness increases with wildlife-friendly garden practices (Hordley and Fox 2024), while trends such as artificial lawns, frequent mowing or weed killer use can reduce available habitat for pollinators (Kirby et al. 2025). Future work integrating habitat quality and management alongside land-cover classes would help to better understand the factors underlying these non-linear relationships.

The overall overlap between approaches was 39 km^2^, or ~ 3% of the study area. While this represents a small proportion of the site, overlapping areas best predicted independent occurrence data—providing evidence that these areas support pollinator movement, and provide clear targets for management when balancing habitat regeneration with other priorities. Non-overlapping areas reflect method-specific sensitivities. For example, GCRGN corridors exclude grasslands smaller than 0.5 ha or near roads, removing smaller or fragmented patches likely captured by species-specific models. Additionally, GCRGN corridors were based on pollinator specialists, while the species-based connectivity approach focussed on pollinator generalists. These differences are reflected in output: GCRGN corridors emphasise connections between species-rich grasslands, and tend to be more frequently associated with impervious surfaces and private gardens, occurring closer to the urban centre. In contrast, species-based connectivity corridors highlight areas aligned with species-habitat preferences, and are more commonly associated with semi-natural habitats such as woodland and wetlands at intermediate distances from the city centre. These non-overlapping areas remain valuable for adding flexibility to corridor design: overlapping areas can form a core network, while non-overlapping areas represent potential extensions. For example, the species-based connectivity model identified regions to the north-west of the study area, dominated by woodland and heather grassland leading towards Loch Lomond and the Trossachs National Park, which were not captured by GCRGN corridors. Similarly, areas to the east show high connectivity around wetland habitat, which are fragmented from the rest of the study area by urban land, and represent locations where GCRGN-predicted corridors could enhance connectivity. Understanding species-habitat relationships can therefore guide interventions to improve functional connectivity. Enhancing habitat quality around pinch points is likely to facilitate movement, and previous research indicates that connectivity of green areas is vital for pollinator richness and abundance in cities (Graffigna et al. 2024). Some pinch points, however, fell within the urban core, where semi-natural habitats may be limited but residential gardens are more abundant. Since private gardens feature prominently in GCRGN corridors, promoting wildlife-friendly gardening practices could be particularly effective in supporting connectivity for urban pollinators (Balogun et al. 2025).

The construction of urban ecological corridors is a long-term process (Peng et al. 2017). The GCRGN grasslands network has already identified over 400 opportunity areas for habitat creation (McLeod 2023). This comparative analysis may help refine priority areas and inform the integration of species-rich grassland corridors, while also providing insights into key habitat types that support insect pollinators and should be prioritised for conservation. Future iterations of the habitat suitability model—incorporating species and habitat data post-establishment of corridors—could help assess their functionality. Since direct validation of connectivity models is challenging, an abundance-based approach, in which pollinator numbers are monitored across the corridor over time, could provide a valuable method for evaluating their effectiveness (Osipova et al. 2019). Collaboration with citizen scientists could enhance this data collection, particularly for rarer, more specialist species, as commonly observed insect groups (e.g., Lepidoptera, Hymenoptera) typically dominate current datasets (Di Cecco et al. 2021). Corridors could be further validated using systematic pollinator transects or by direct observations of pollinator movements along corridors. Additionally, modelling species-specific connectivity (e.g., by splitting up pollinators into functional assemblages: Prima et al. 2024) could improve model predictions, though processing constraints remain a challenge. For example, circuit theory and pinch point computational time depends on node/core area delineation, and calculating connectivity between many core areas of habitat can take up to several days to compute. Finally, when planning and evaluating corridors, methodological factors such as the artificial delineation of study boundaries can introduce edge effects that distort connectivity estimates, potentially inflating landscape resistance (Koen et al. 2010). Using a buffer area can help minimise these effects, particularly near urban zones, which was the focus of our study. In this analysis, any potential border effects were likely minimal, as high connectivity values were distributed across the study area, from interior regions to the edges.

This study demonstrates how different multi-species approaches can enhance ecological connectivity analyses and inform urban wildlife corridor planning. While our results are not generalisable—given they are based on a specific location, habitat type, and set of species—the essence of our combined approach, integrating different analytical methods to assess multi-species connectivity, can be applied to other contexts. No single analytical approach exists for effective connectivity conservation planning (Keeley et al. 2019) and therefore validating corridors using complementary methods can improve their robustness and practical applicability (Zeller et al. 2012). For both methods the use of biological records was intrinsic to developing nature networks. Although conservation planners often rely solely on environmental or landcover data, these can be poor surrogates for biodiversity (Mossman et al. 2015). Biological records, however, can represent an important resource that could be harnessed within city planning. Incorporating connectivity modelling into planning frameworks requires collaboration and a shared vision between multiple stakeholders, such as ecologists, planners, policymakers, and local communities (Keeley et al. 2018). In our case, though collaboration comes with its own challenges (such as data resolution mismatches or differences in policy timescales), working with Green Network Development Officers facilitates direct communication about potential gaps in their network. Ensuring that outputs can feed directly into local authority planning software and GIS systems is also critical for supporting decision-making. Other research has developed interactive tools to allow planners to visualise how urban development or habitat creation can influence wildlife corridors for particular species (Schneider et al. 2019; Nelli et al. 2022). Expanding such approaches to multiple taxonomic groups (e.g., birds, mammals, amphibians) is the next step in designing corridors that enhance wildlife movement in urban landscapes. Other pathways for translating predicted nature networks into on-the-ground action could include submission to planning authorities, for example, Glasgow City Council’s City Development Plans (Glasgow City Council 2024b) or Local Place Plans (Glasgow City Council 2025), which allow communities to submit proposals for land development projects. Ultimately, although combining methods for estimating ecological corridors involves trade-offs (e.g., differences in spatial or temporal scale), our study highlights how considering multiple approaches not only improves our understanding of taxa-specific corridors within urban environments, but also increases confidence in their implementation, leading to more effective conservation outcomes.

## Supplementary Information

Below is the link to the electronic supplementary material.

Supplementary file1 (DOCX 404 KB)

## Data Availability

Datasets used in this study were obtained from publicly available sources, including biological records and spatial datasets. No new datasets were generated for this study.
