# Supplementary material for: Designing nature networks for cities: combining multi-species modelling approaches
Source: Landsc Ecol. 2026 Mar 1;41(3):56. doi: 10.1007/s10980-026-02315-0 (PMC12982267; doi:10.1007/s10980-026-02315-0)
Supplement: Supplementary file 1 — Supplementary file1 (DOCX 404 KB) [file 10980_2026_2315_MOESM1_ESM.docx]

**Supplementary Material *for***

**Designing nature networks for cities:**

**combining multi-species modelling approaches**

Table of Contents

[Supplementary figures 2](#_Toc218609731)

[Figure S1 2](#_Toc218609732)

[Figure S2 3](#_Toc218609733)

[Figure S3 4](#_Toc218609734)

[Figure S4 5](#_Toc218609735)

[Supplementary tables 7](#_Toc218609736)

[Table S1 7](#_Toc218609737)

[Table S2 8](#_Toc218609738)

[Table S3 9](#_Toc218609739)

[Table S4 10](#_Toc218609740)

[Table S5 10](#_Toc218609741)

[Table S6 11](#_Toc218609742)

[References 12](#_Toc218609743)

# Supplementary figures


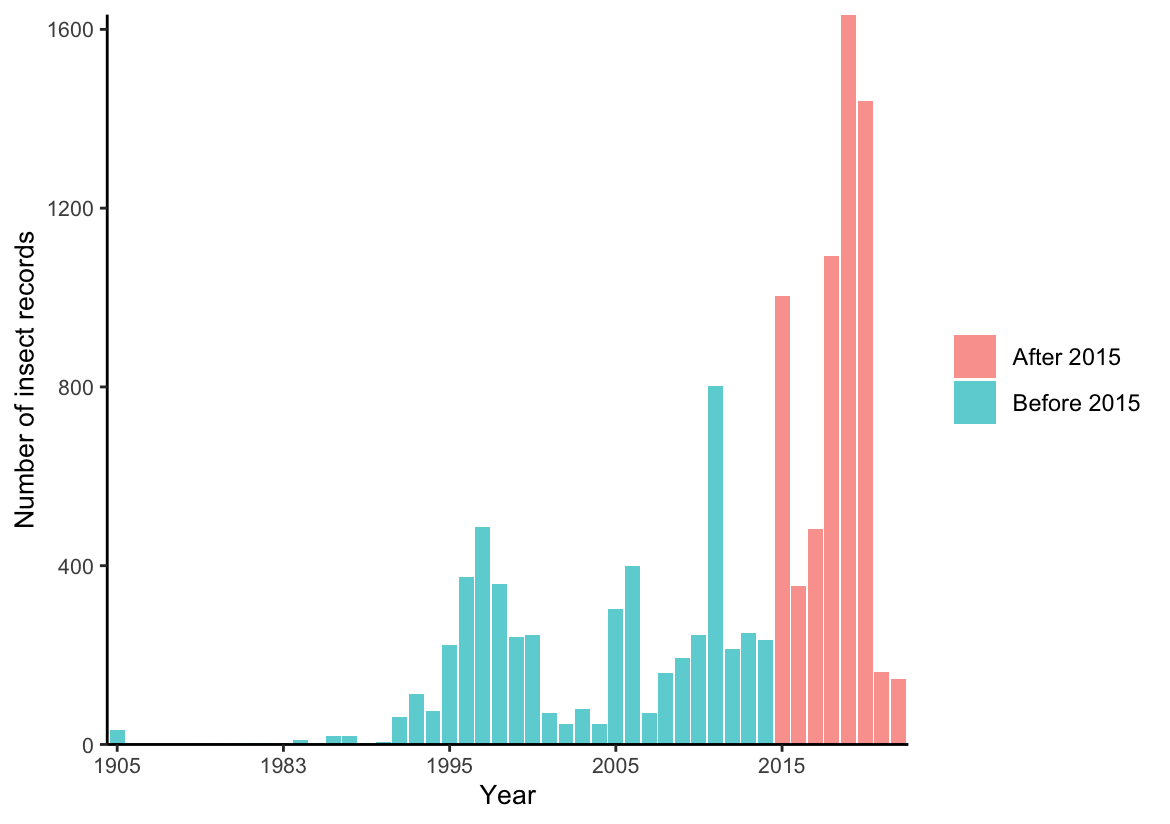


Figure S1. Distribution of pollinator occurrence records, before and after the year 2015. Provided by Glasgow Biological Records Centre.


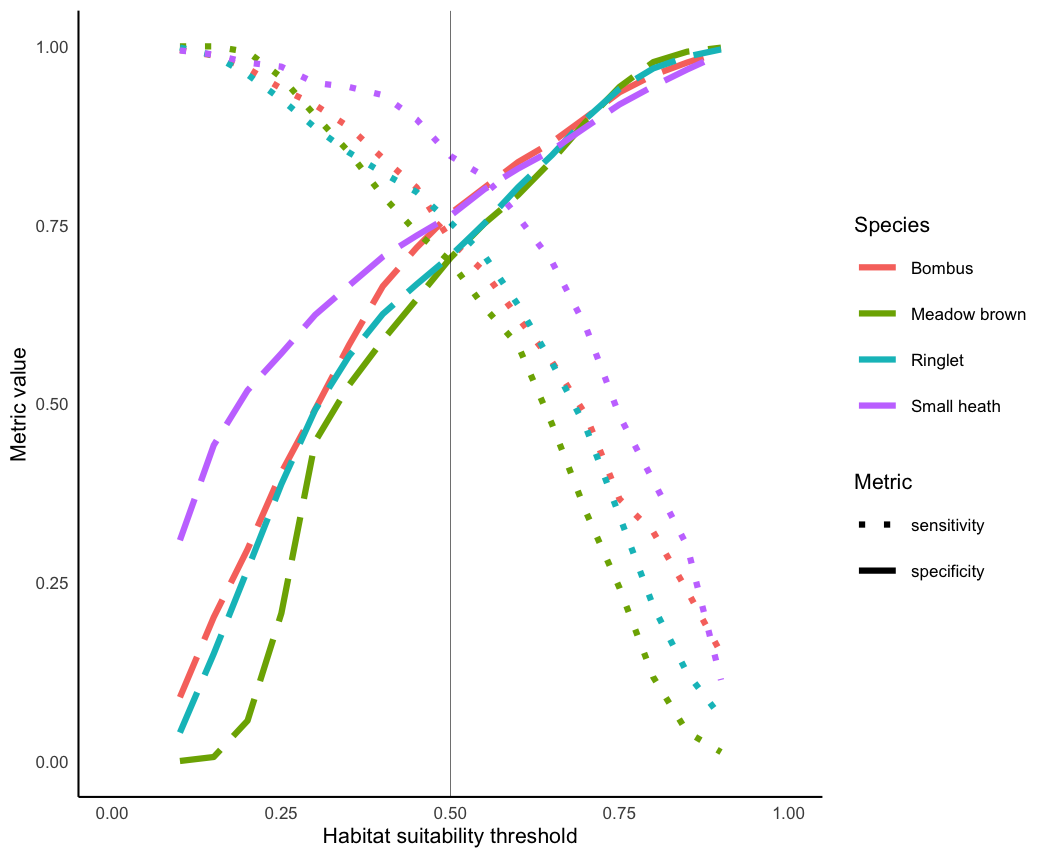


Figure S2. Sensitivity and specificity of individual pollinator models across different habitat suitability thresholds. Thin vertical lines indicate core area threshold (0.5). Long dashed lines represent specificity scores and dotted lines sensitivity scores. Species are represented using different colours.


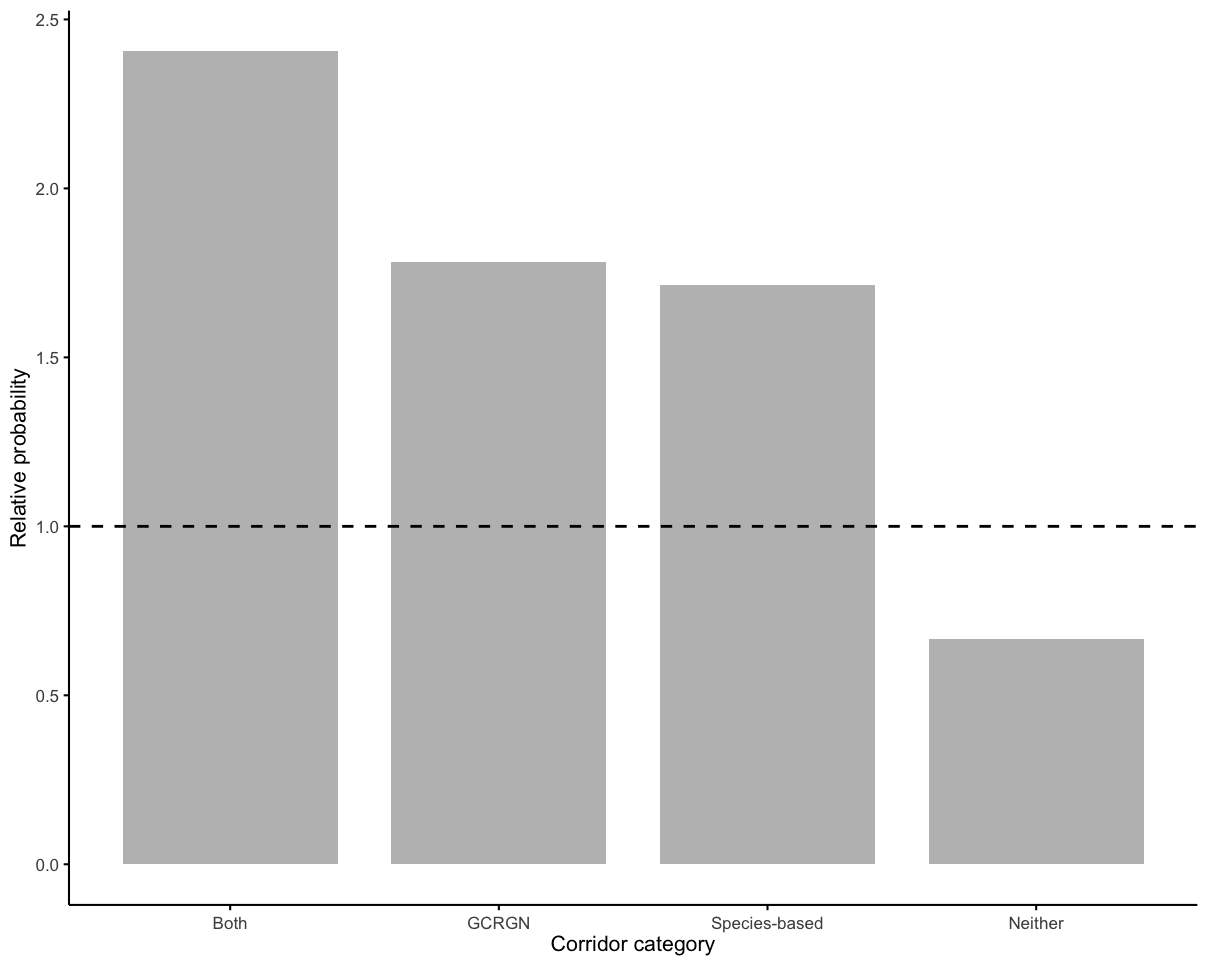


Figure S3. Relative probability of independent GBIF presence locations falling in each corridor category: both, only GCRGN corridors, only species-based corridors (top 10% of circuitscape values), or neither corridor type. Relative probability is the proportion of GBIF records in each category divided by the proportion of background records in each category. The horizontal dashed line at 1 represents random expectation. Values above 1 indicate GBIF presences are more likely than random to occur in that category, while values below 1 indicate lower probability than random.


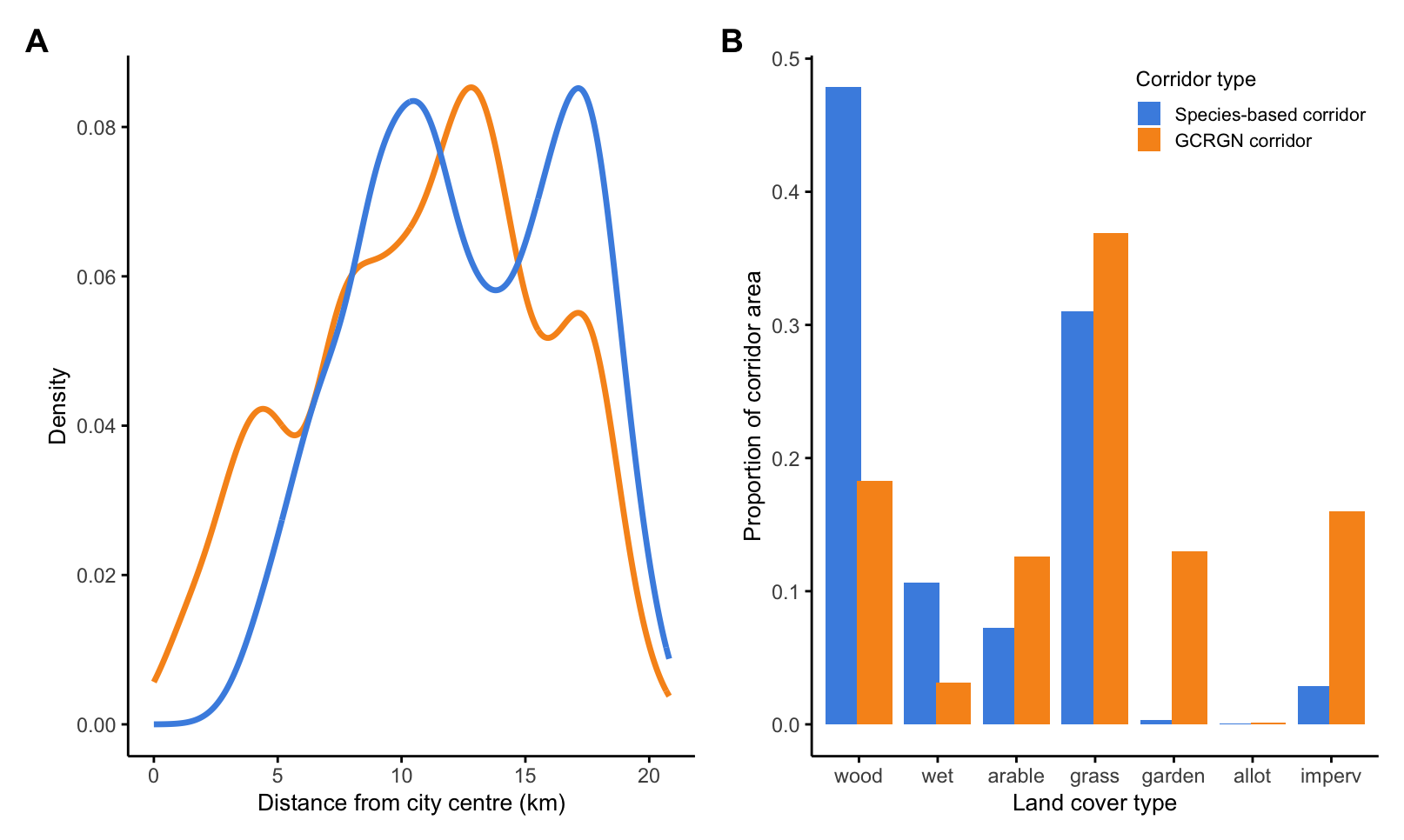


Figure S4. Descriptive statistics of where each corridor type is predicted to occur. (A) Density histogram of corridor distances (km) to Glasgow city centre. Distances are calculated for grid cells containing corridors predicted by either species-based (blue) or GCRGN (orange) models. (B) Overall proportion of landcover classes in species-based (blue) or GCRGN (orange) corridors. Descriptive statistics are calculated for non-overlapping areas where only one corridor type is predicted to occur.


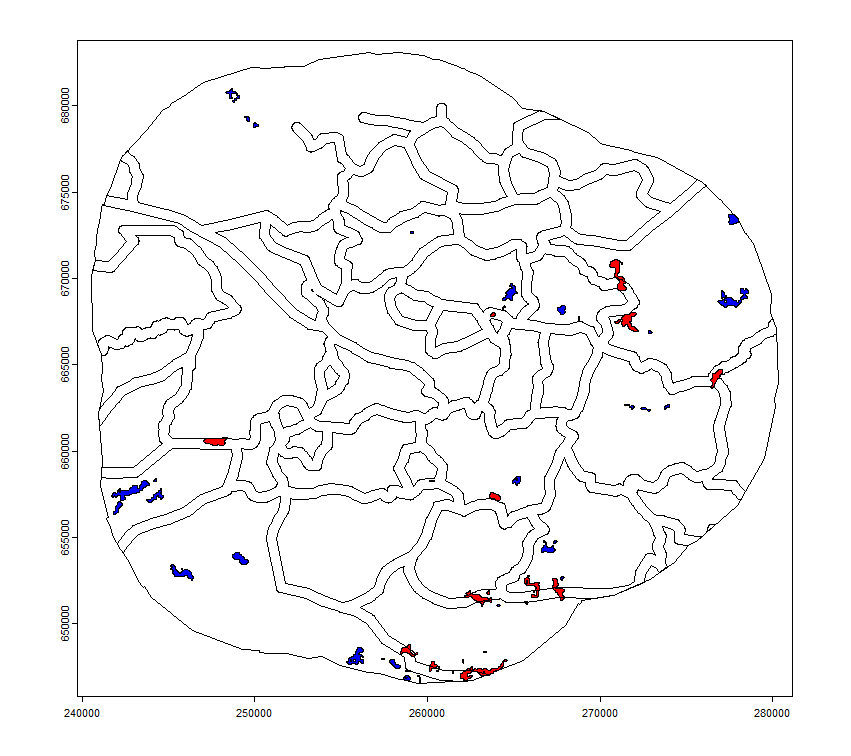
Figure S5. Overlap (red) and gaps (blue) of pinch points (areas in top 1% of constrained movement) for pollinator species in Glasgow, UK. Pinch points were determined from inverted habitat suitability maps. GCRGN corridors are indicated by black outline.

# Supplementary tables

Table S1. Characteristics of four candidate pollinator species

|  | Species latin name | Species common name | Typical dispersal distance | Occurrence |
| --- | --- | --- | --- | --- |
| Bees | *Bombus* spp | White/buff-tailed bumblebee | 300-700 m (Knight et al., 2005; Wolf & Moritz, 2008) | 390 |
| Butterflies | *Maniola jurtina* | Meadow brown | 100-300 m (Ouin et al., 2008; Villemey et al., 2016) | 392 |
|  | *Aphantopus hyperantus* | Ringlet | 100 – 500 m (Billeter et al., 2003; Polic et al., 2014) | 633 |
|  | *Coenonympha pamphilus* | Small heath | 100 – 400 m (Wickman, 1985) | 176 |

Table S2. Spearman correlation matrix of predictor variables used in habitat suitability modelling. Strong correlations (*rs* > 0.7) are highlighted in bold.

|  | Wood | Wet | Arable | Grass | Garden | Allot | Imperv | Alt | Pop  Density |
| --- | --- | --- | --- | --- | --- | --- | --- | --- | --- |
| Wood | 1 | 0.05 | -0.00 | 0.01 | -0.29 | 0.01 | -0.31 | -0.00 | -0.23 |
| Wet |  | 1 | -0.02 | 0.13 | -0.32 | -0.03 | -0.33 | 0.31 | -0.35 |
| Arable |  |  | 1 | -0.04 | -0.19 | -0.00 | -0.12 | -0.31 | -0.07 |
| Grass |  |  |  | 1 | -0.57 | -0.05 | -0.67 | 0.52 | -0.65 |
| Garden |  |  |  |  | 1 | 0.04 | **0.76** | -0.32 | **0.82** |
| Allot |  |  |  |  |  | 1 | 0.06 | -0.06 | 0.06 |
| Imperv |  |  |  |  |  |  | 1 | -0.43 | **0.78** |
| Alt |  |  |  |  |  |  |  | 1 | -0.53 |
| Pop  Density |  |  |  |  |  |  |  |  | 1 |

Table S3. Comparison of generalised additive models using AIC, testing each of three correlated urban predictors (proportion of gardens, proportion impervious surfaces and human population densities) on pollinator occurrence in Glasgow, UK. All predictor variables were scaled. Lower AIC indicates better model support.

| Species | Variable | AIC |
| --- | --- | --- |
| Bombus | s(garden)  s(imperv)  s(popdensity) | 469.0114  474.0456  473.2369 |
| Meadow brown | s(garden)  s(imperv)  s(popdensity) | 494.3850  491.2663  495.0522 |
| Ringlet | s(garden)  s(imperv)  s(popdensity) | 743.8404  739.1682  748.0072 |
| Small heath | s(garden)  s(imperv)  s(popdensity) | 203.1243  203.9216  202.1869 |

Table S4. Habitat suitability thresholds which maximised the sum of sensitivity and specificity (Max SSS: Liu et al., 2013) and minimised the sensitivity-specificity difference (“minimised difference threshold” or MDT: Jiménez-Valverde & Lobo, 2007), for generalised additive models assessing pollinator occurrence in Glasgow, UK. Results are from a sensitivity analysis assessing model discrimination across various habitat suitability thresholds (from 0.1 – 0.95).

| Species | Metric | Metric Value | Habitat Suitability Threshold |
| --- | --- | --- | --- |
| Bombus | Max SSS | 1.521 | 0.45 |
|  | MDT | 0.037 | 0.5 |
| Meadow brown | Max SSS | 1.402 | 0.5 |
|  | MDT | 0.005 | 0.5 |
| Ringlet | Max SSS | 1.463 | 0.45 |
|  | MDT | 0.044 | 0.55 |
| Small heath | Max SSS | 1.637 | 0.4 |
|  | MDT | 0.018 | 0.55 |

Table S5. Comparison of performance metrics from random and spatially blocked 10-fold cross validation of generalised additive models examining the effect of environmental variables on pollinator presence in Glasgow, UK.

| Species | Model assessment | | | | | |
| --- | --- | --- | --- | --- | --- | --- |
|  | AUC | | Boyce Index | | TSS | |
|  | Random | Spatial | Random | Spatial | Random | Spatial |
| Bombus | 0.79 | 0.71 | 0.93 | 0.63 | 0.46 | 0.36 |
| Meadow brown | 0.74 | 0.7 | 0.88 | 0.58 | 0.38 | 0.28 |
| Ringlet | 0.79 | 0.71 | 0.97 | 0.8 | 0.44 | 0.31 |
| Small heath | 0.85 | 0.73 | 0.91 | 0.55 | 0.55 | 0.34 |

Table S6. Model output of smoothed terms from final selected models when examining effect of environmental covariates on pollinator occurrence in Glasgow, UK. Smoothed terms were subject to “shrinkage” for model selection. All predictor variables were scaled in models.

| Bombus spp | | | | |
| --- | --- | --- | --- | --- |
| Term | Estimated df* | Reference df* | Chi-square | p-value |
| s(sqrt(alt)) | 2.678 | 4 | 5.162 | 0.066 |
| s(garden) | 2.605 | 4 | 19.850 | <0.001 |
| s(allot) | 0.000 | 4 | 0.000 | 0.406 |
| s(wood) | 2.925 | 4 | 43.421 | <0.001 |
| s(wet) | 1.231 | 4 | 10.724 | <0.001 |
| s(grass) | 2.764 | 4 | 7.102 | <0.05 |
| s(arable) | 0.000 | 4 | 0.000 | 0.819 |
| s(x,y) | 22.45 | 29 | 50.449 | <0.01 |
| Meadow brown | | | | |
| Term | Estimated df* | Reference df* | Chi-square | p-value |
| s(sqrt(alt)) | 0.965 | 4 | 1.459 | 0.139 |
| s(garden) | 0.038 | 4 | 0.049 | 0.235 |
| s(allot) | 0.000 | 4 | 0.000 | 0.578 |
| s(wood) | 1.999 | 4 | 61.826 | <0.001 |
| s(wet) | 3.003 | 4 | 27.274 | <0.001 |
| s(grass) | 1.426 | 4 | 5.230 | <0.05 |
| s(arable) | 2.724 | 4 | 13.358 | <0.01 |
| s(x,y) | 7.583 | 29 | 13.699 | <0.05 |
| Ringlet | | | | |
| Term | Estimated df* | Reference df* | Chi-square | p-value |
| s(sqrt(alt)) | 2.501 | 4 | 9.608 | <0.01 |
| s(garden) | 2.629 | 4 | 20.200 | <0.001 |
| s(allot) | 0.000 | 4 | 0.000 | 0.991 |
| s(wood) | 3.492 | 4 | 73.674 | <0.001 |
| s(wet) | 3.210 | 4 | 24.484 | <0.001 |
| s(grass) | 1.972 | 4 | 7.840 | <0.01 |
| s(arable) | 0.184 | 4 | 0.199 | 0.315 |
| s(x,y) | 13.21 | 29 | 28.943 | <0.01 |
| Small heath | | | | |
| Term | Estimated df* | Reference df* | Chi-square | p-value |
| s(sqrt(alt)) | 0.077 | 4 | 0.090 | 0.243 |
| s(garden) | 0.897 | 4 | 10.174 | <0.001 |
| s(allot) | 0.000 | 4 | 0.000 | 0.786 |
| s(wood) | 2.403 | 4 | 25.040 | <0.001 |
| s(wet) | 2.457 | 4 | 24.332 | <0.001 |
| s(grass) | 0.000 | 4 | 0.000 | 0.795 |
| s(arable) | 0.577 | 4 | 1.145 | 0.149 |
| s(x,y) | 24.06 | 29 | 55.655 | <0.001 |

*df = degrees of freedom

# References

Billeter, R., Sedivy, I., & Diekotter, T. (2003). Distribution and dispersal patterns of the ringlet butterfly (Aphantopus hyperantus) in an agricultural landscape. *Bulletin of the Geobotanical Institute ETH, 69*, 45-55.

Jiménez-Valverde, A., & Lobo, J. M. (2007). Threshold criteria for conversion of probability of species presence to either–or presence–absence. *Acta Oecologica, 31*, 361-369.

Knight, M. E., Martin, A. P., Bishop, S., Osborne, J. L., Hale, R. J., Sanderson, R. A., & Goulson, D. (2005). An interspecific comparison of foraging range and nest density of four bumblebee (*Bombus*) species. *Molecular Ecology, 14*, 1811-1820.

Liu, C., White, M., & Newell, G. (2013). Selecting thresholds for the prediction of species occurrence with presence‐only data. *Journal of Biogeography, 40*, 778-789.

Ouin, A., Martin, M., & Burel, F. (2008). Agricultural landscape connectivity for the meadow brown butterfly (*Maniola jurtina*). *Agriculture, Ecosystems & Environment, 124*, 193-199.

Polic, D., Fiedler, K., Nell, C., & Grill, A. (2014). Mobility of ringlet butterflies in high-elevation alpine grassland: effects of habitat barriers, resources and age. *Journal of Insect Conservation, 18*, 1153-1161.

Villemey, A., Peterman, W. E., Richard, M., Ouin, A., Van Halder, I., Stevens, V. M., Baguette, M., Roche, P., & Archaux, F. (2016). Butterfly dispersal in farmland: a replicated landscape genetics study on the meadow brown butterfly (*Maniola jurtina*). *Landscape Ecology, 31*, 1629-1641.

Wickman, P.-C. (1985). The influence of temperature on the territorial and mate locating behaviour of the small heath butterfly, *Coenonympha pamphilus* (L.)(Lepidoptera: Satyridae). *Behavioral Ecology and Sociobiology, 16*, 233-238.

Wolf, S., & Moritz, R. F. (2008). Foraging distance in *Bombus terrestris* L.(Hymenoptera: Apidae). *Apidologie, 39*, 419-427.
